# High-dose-rate brachytherapy in uterine cervix carcinoma: a comparison of dosimetry and clinical outcomes among three fractionation schedules

**DOI:** 10.3389/fonc.2024.1366323

**Published:** 2024-07-12

**Authors:** Haiyan Wu, Yanan He, Duke Chen, Mei Liu, Xiujuan Zhao

**Affiliations:** ^1^ Department of Radiation Oncology, Chongqing University Cancer Hospital, Chongqing, China; ^2^ Department of Gynecological Oncology, Chongqing University Cancer Hospital, Chongqing, China

**Keywords:** fractionation schedule, brachytherapy, cervical cancer, dosimetry comparison, clinical outcome

## Abstract

**Background:**

To assess the differences among three dose-fractionation schedules of image-guided adaptive brachytherapy (IGABT) in cervical squamous cell carcinoma (CSCC) by comparing the dosimetry and clinical outcomes.

**Methods:**

Forty-five patients with CSCC who underwent chemoradiotherapy and IGABT were retrospectively enrolled and divided into three groups based on their dose-fractionation schedules of brachytherapy as: Group-5.5 (5.5 Gy × 6 fractions), Group-6.0 (6.0 Gy × 5 fractions), and Group-7.0 (7.0 Gy × 4 fractions). The analyzed dose-volume histogram parameters included D_90%_ and D_98%_ of the high-risk clinical target volume (HR-CTV), D_90%_ and D_98%_ of intermediate-risk clinical target volume (IR-CTV), and D_0.1cc_ and D_2cc_ of the organs-at-risk (OARs, namely the bladder, rectum, sigmoid and small intestine). Furthermore, the therapeutic efficacy and late toxicities were also compared among the three groups.

**Results:**

The doses of HR-CTV and IR-CTV in Group-5.5 were found to be the highest among the three groups, followed by those in Group-6.0. Significant differences were found for the doses of HR-CTV between Group-5.5 and the other groups. There were no significant differences in the bladder, sigmoid and small intestine dose among the three groups. However, Group-6.0 yielded the lowest rectum received doses, with a significant difference in D_0.1cc_ being detected between Group-6.0 and Group-5.5. The median follow-up time was 30.08 months [range, 6.57–46.3]. The numbers of patients with complete response in Group-5.5, Group-6.0 and Group-7.0 were 13, 14 and 14, respectively (P > 0.05). In regard to the toxicitiy, the incidence of radiation cystitis and proctitis in Group-6.0 was lower than that in Group-5.5 and Group-7.0 (P > 0.05).

**Conclusions:**

The dose-fractionation schedule of 6.0 Gy × 5 fractions provided the most beneficial effects with relatively low OARs doses, suggesting that this dose-fractionation schedule should be prioritized in the clinical application of brachytherapy in cervical cancer.

## Introduction

Cervical cancer (CC) is a major cause of morbidity and mortality among women, with approximately 604,000 newly diagnosed cases and 342,000 deaths in 2020 (1). Brachytherapy plays a critical role in the treatment of locally advanced CC, providing significant benefits for the improved pelvic control and overall survival (2). The efficacy of brachytherapy is largely associated with the total doses and dose per fraction, which may induce short and long term injury to normal tissues. The American Brachytherapy Society has recommended that brachytherapy with doses ranging from 4 to 8 Gy per fraction, administered in 4 to 8 fractions, is feasible in clinical practice (3). In accordance with the current guidelines and recommendations, several dose-fractionation schedules, such as 30 Gy in 5 fractions (6 Gy × 5 F) and 28 Gy in 4 fractions (7 Gy × 4 F), have been widely used in brachytherapy. However, there is a lack of consensus in the choice of dose-fractionation schedule considering the current workflow. Indeed, a variety of the schedules are practiced in different institutions, and even within the same institution, variations exist due to different opinions of physicians (4–14). Therefore, there is a need to find a better schedule that allows reducing normal tissue complications without compromising local control of the disease among the schedules commonly used in clinical practice.

In most of the existing studies, the prescribed dose refers to the dose at point A, which is the point located at 2 cm lateral to the central canal of the uterus and 2 cm from the mucous membrane of the lateral fornix in the axis of the uterus (15). However, this approach is not suitable for tumors that are too large, too small, or irregularly shaped, and the prescribed dose at point A cannot correctly represent the dose received by the tumors. Furthermore, this can also increase the dose received in the organs-at-risk (OARs), such as the rectum (16). Dose assessment of tumors and OARs in image-guided adaptive brachytherapy (IGABT) has gradually changed from point dose to dose-volume parameters, thereby improving the accuracy of dose assessment and the underlying treatment (17–19). In our institution, three dose-fractionation schedules are routinely used in brachytherapy, based on the dose-volume parameters. Therefore, the aim of this study was to provide clinical evidence for the selection of dose-fractionation schedules in IGABT, by comparing the differences between the three schedules in terms of dosimetric parameters, therapeutic efficacy, and late toxicities.

## Methods

The study protocol was approved by the medical research ethical committee of our institutional review board. Written informed consent was waived, considering the retrospective nature of this study that the entire analysis was based on existing clinical data, and did not interfere with the current clinical workstream.

### Patient characteristics

A total of 66 patients with cervical squamous cell carcinoma (CSCC) who underwent brachytherapy from May 2020 to December 2021 were screened from our institution’s database. Among them, 21 patients who met the following criteria were excluded: (a) Incomplete the courses of radiotherapy, e.g., brachytherapy or intensity modulated radiotherapy (IMRT), as planned (N = 5); (b) Inability for follow-up (N = 12); and (c) History of pelvic radiotherapy (N = 4). Finally, 45 patients were identified and categorized into three groups based on their dose-fractionation schedules of brachytherapy: Group-5.5 (5.5 Gy × 6 F), Group-6.0 (6.0 Gy × 5 F), and Group-7.0 (7.0 Gy × 4 F), with each group consisting of 15 patients. The staging of CC for each patient was determined in accordance with the staging criteria of the International Federation of Gynecology and Obstetrics (2018) (20).

### Concurrent chemoradiotherapy

In this study, all patients received pelvic IMRT with the same dose of 45Gy in 25 fractions. An IMRT boost to pelvic lymph nodes was allowed, with total lymph node doses boosted to 55–60Gy. To avoid prolonging the total treatment duration, brachytherapy was started only in the middle and late stages of IMRT. The brachytherapy sessions were conducted once a week during the IMRT schedule without overlapping IMRT, while twice a week after the completion of IMRT. During the course of radiotherapy, single-agent cisplatin chemotherapy was administered weekly at a dose of 40 mg/m^2^ (5–6 cycles in total).

### Brachytherapy

All patients underwent high-dose-rate brachytherapy with Iridium-192 (^192^Ir) remote after-loading system. Before implantation of the applicators, patients were counseled to empty bowel. The intracavitary applicator, namely Fletcher Williamson Asia Pacific applicator set, was routinely used in clinical settings. Additional interstitial needles were employed when the dose distribution failed to meet clinical requirements for tumors with large or irregular shape. Subsequently, a gauze was then packed at both the anterior and posterior to push away the bladder, rectum and fix the applicator in place. A computed tomography (CT) scan was performed with the patient in a lithotomy position, covering the area from the plane of the iliac spine to 2 cm below the vaginal opening, with a slice thickness of 3 mm. The position of the applicator set was scrutinized and, if necessary, adjusted according to the scanned images.

**Figure 1:**
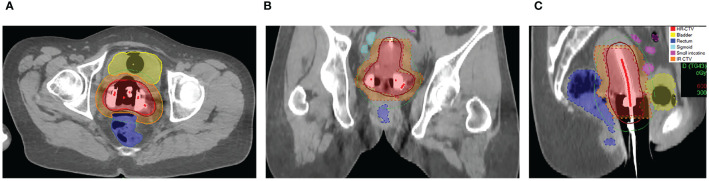
Dose distribution after plan optimization using the prescription dose of 6.0 Gy (**A**: axial image; **B**: coronal image; **C**: sagittal image).

**Figure 2:**
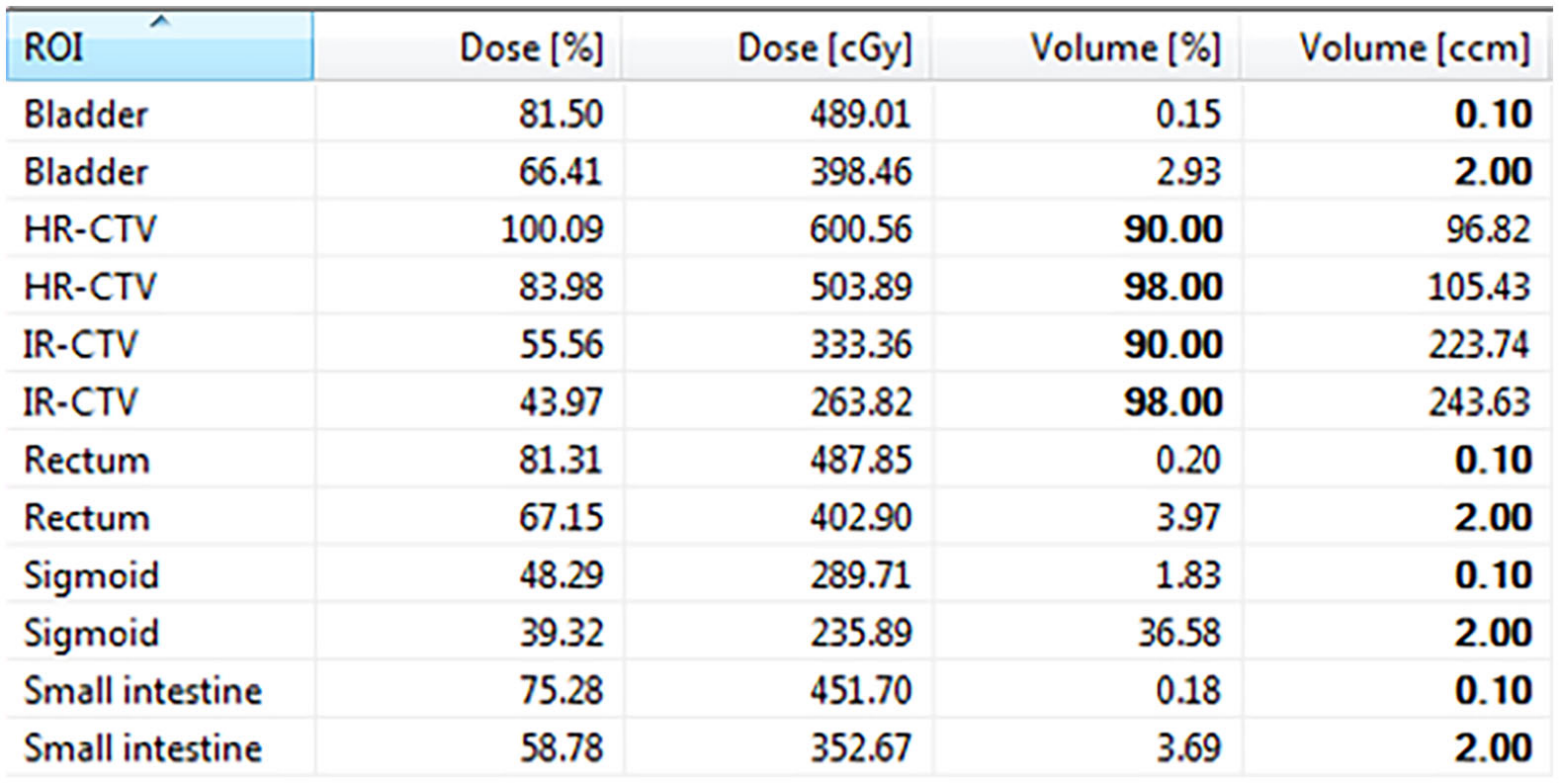
Dose-volume histograms parameters.

Consequently, the CT images were transferred to the Oncentra Brachy treatment planning system (version 4.6.0, Elekta). HR-CTV, IR-CTV and OARs, including the bladder, rectum, sigmoid, and small intestine were delineated on CT images with reference to available information (e.g., gynecological examination and pre-brachytherapy magnetic resonance images). The delineation was performed in accordance with the *International Commission on Radiation Units (ICRU) Report 89* and *IBS-GEC ESTRO-ABS Recommendations* (21). Prescription doses at HR-CTV for patients in Group-5.5, Group-6.0, and Group-7.0 were 5.5 Gy, 6.0 Gy, and 7.0 Gy, respectively. The dose received by at least 90% (D_90%_) of HR-CTV and the minimum dose in the most irradiated 2 cm^3^ volume (D_2cc_) in the OARs were used as the constraints to determine the optimal treatment plan by graphical optimization or manual adjustment of the source dwell patterns (e.g., times and positions). With the prescription dose of 6.0 Gy as an example, the dose distribution after plan optimization and the corresponding dose-volume histograms parameters of the plan are shown in **Figure 1** and **Figure 2**, respectively.

### Evaluation

For dosimetric evaluation, the D_90%_ and D_98%_ of HR-CTV, the D_90%_ and D_98%_ of IR-CTV, and the D_0.1cc_, D_2cc_ of OARs were calculated. The brachytherapy doses were then converted into the equivalent doses in 2 Gy/F (EQD_2_), as recommended by the ICRU Report 89. The EQD_2_ was calculated using the following formula: EQD_2_= nd × (α/β + d)/(α/β + 2), where d represents the dose per fraction, n is the number of fractions, the value of α/β is 10 for HR-CTV, and the value of α/β is 3 for OARs (15). Therapeutic efficacy was assessed based on the complete response (CR) and partial response (PR), as defined in the *Response Evaluation Criteria in Solid Tumor Guidelines* (version 1.1). Late toxicities were evaluated by assessing the incidences of radiation cystitis and proctitis in the three groups, according to the radiation toxicity grading criteria of the Radiation Therapy Oncology Group (RTOG) and the European Organization for Research and Treatment of Cancer (EORTC).

### Statistical analysis

All statistical analyses were performed using IBM SPSS Statistics (IBM SPSS Statistics for Macintosh, Version 26.0, IBM, Armonk, NY, USA). Continuous variables were presented as mean ± standard deviation, and the categorical variables were presented as counts and percentages. The Kruskal–Wallis test was used to analyze the patient information and EQD_2_ of HR-CTV, IR-CTV and OARs in the three groups, and the Wilcoxon test was used for *post-hoc* multiple comparisons. The count data were analyzed by chi-square test or rank sum test, according to the data distribution. P < 0.05 was considered to indicate a statistically significant difference.

## Results

The detailed information of patient characteristics is presented in **Table 1**. Among the 45 patients included in this study, a total of 225 brachytherapy plans were analyzed, comprising 90 plans in Group-5.5, 75 plans in Group-6.0, and 60 plans in Group-7.0. There were no significant differences in terms of the patient’s age, staging, hemoglobin level, squamous cell carcinoma antigen level, volume of HR-CTV at the first time of brachytherapy, the overall treatment time, the proportion of intracavitary/interstitial brachytherapy, and the number of needles used in brachytherapy, between the three groups (all P > 0.05).

**Table 1:** Patient characteristics.

Characteristics	Group-5.5	Group-6.0	Group-7.0	P value
**Age (years)**	62.47 ± 8.58 (48–77)	55.2 ± 10.5 (33–76)	56.8 ± 7.86 (49–73)	0.620
**FIGO stage (2018)**				0.546
I	0	1 (6.67%)	0	
II	6 (40.00%)	7 (46.67%)	6 (40.00%)	
III	8 (53.33%)	7 (46.67%)	9 (60.00%)	
IV	1 (6.67%)	0	0	
**Hemoglobin (g/L)**	104.07 ± 12.77 (81–121)	108.47 ± 14.55 (83–13)	103.47 ± 11.99 (78–120)	0.571
**SCC (ng/mL)**	6.60 ± 8.82 (0.3–28.1)	6.63 ± 9.57 (0.5–30.3)	8.07 ± 10.90 (0.9–34.5)	0.490
**Volume of HR-CTV(cm^3^)**	50.84 ± 20.7	48.89 ± 18.48	51.24 ± 20.46	0.945
**OTT (days)**	59.47 ± 3.58	56.40 ± 3.60	55.20 ± 6.74	0.053
**Intracavitary/interstitial brachytherapy**	31 (34.44%)	26 (34.67%)	20 (33.33%)	0.985
**Number of needles**	1.58 ± 0.67 (1–4)	1.69 ± 0.47 (1–2)	1.85 ± 0.37 (1–2)	0.938

FIGO, International Federation of Gynecology and Obstetrics; SCC, Squamous Cell Carcinoma Antigen; OTT, Overall Treatment Time.

### Dosimetric analysis

**Figure 3:**
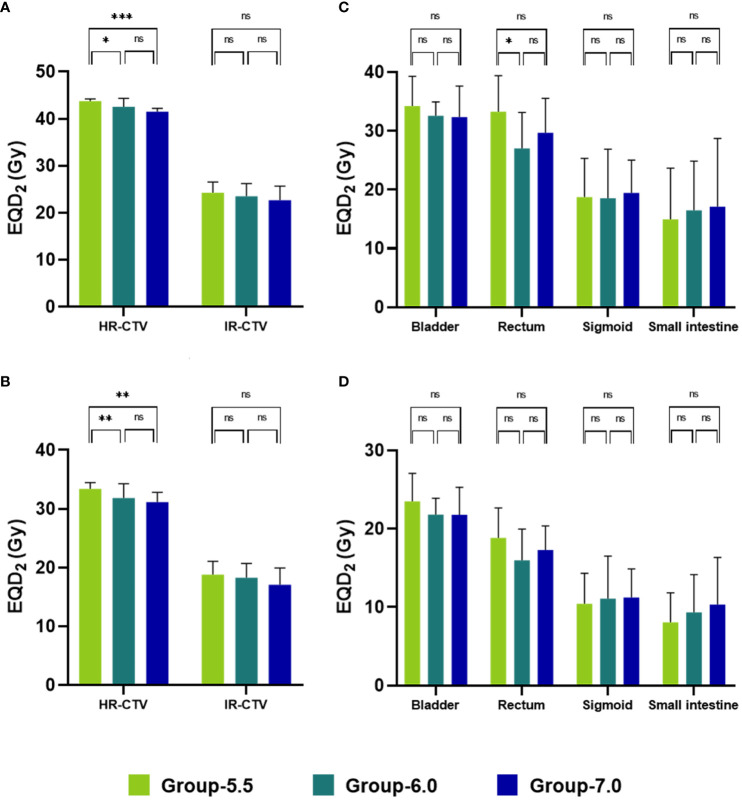
Mean EQD_2_ of HR-CTV, IR-CTV and OARs of the three dose-fractionation schedules (**A**: D_90%_; **B**: D_98%_; **C**: D_0.1cc_; **D**: D_2cc_). *, *P* < 0.05; **, *P* < 0.01; ***, *P* < 0.001; ns, *P* > 0.05.

The doses of HR-CTV in Group-5.5 were significantly higher than those in Group-6.0 (D_90%_: 43.79 ± 0.45 Gy vs. 42.57 ± 1.8 Gy, P = 0.018; D_98%_: 33.43 ± 1.07 Gy vs. 31.84 ± 2.45 Gy, P = 0.008) and Group-7.0 (D_90%_: 43.79 ± 0.45 Gy vs. 41.54 ± 0.7 Gy, P < 0.001; D_98%_: 33.43 ± 1.07 Gy vs. 31.15 ± 1.68 Gy, P = 0.003). The doses of IR-CTV were also found to be the highest in Group-5.5, while no significant differences were found among the three groups. The doses of HR-CTV and IR-CTV in Group-7.0 were found to be the lowest among the three groups. Comparison of the OAR doses in the three groups showed that the rectum and bladder doses in Group-5.5 were higher than those in Group-6.0 and Group-7.0. The rectum D_0.1cc_ in Group-5.5 was significantly higher than that in Group-6.0 (33.29 ± 6.1 Gy vs. 27.03 ± 6.12 Gy, P = 0.024), whereas the sigmoid D_2cc_ and small intestine D_2cc_ in Group-5.5 was the lowest among the three groups. The rectum, sigmoid and small intestine doses of Group-6.0 were lower than those of Group-7.0 (**Table 2**, **Figure 3**).

**Table 2:** Total EQD_2_ of HR-CTV, IR-CTV and OARs with different dose prescriptions.

Dose (Gy)	Group-5.5	Group-6.0	Group-7.0	P value
HR-CTV				
D_90%_	43.79 ± 0.45	42.57 ± 1.8#	41.54 ± 0.7*	<0.001
D_98%_	33.43 ± 1.07	31.84 ± 2.45#	31.15 ± 1.68*	0.001
IR-CTV				
D_90%_	24.29 ± 2.29	23.51 ± 2.69	22.67 ± 3.04	0.231
D_98%_	18.81 ± 2.28	18.29 ± 2.43	17.08 ± 2.87	0.131
Bladder				
D_0.1cc_	34.24 ± 5.03	32.59 ± 2.37	32.35 ± 5.29	0.834
D_2cc_	23.5 ± 3.58	21.8 ± 2.11	21.79 ± 3.52	0.340
Rectum				
D_0.1cc_	33.29 ± 6.1	27.03 ± 6.12#	29.68 ± 5.87	0.029
D_2cc_	18.83 ± 3.82	15.98 ± 3.99	17.31 ± 3.04	0.118
Sigmoid				
D_0.1cc_	18.76 ± 6.57	18.56 ± 8.33	19.46 ± 5.56	0.873
D_2cc_	10.43 ± 3.91	11.08 ± 5.46	11.24 ± 3.65	0.888
Small intestine				
D_0.1cc_	16.54 ± 7.29	16.50 ± 8.34	17.13 ± 11.60	0.971
D_2cc_	8.51 ± 3.30	9.34 ± 4.82	10.34 ± 6.02	0.898

# P<0.05 (Group-6.0 vs. Group-5.5).

* P<0.05 (Group-7.0 vs. Group-5.5).

### Therapeutic efficacy and toxicity

In terms of therapeutic efficacy, the CR rate in Group-6.0 was comparable to that in Group-7.0 (93.33%), and higher than that in Group-5.5 (93.33% vs. 86.67%) without statistical difference (**Table 3**). In terms of radiation cystitis, only Grade 2 radiation cystitis was found in all group. The incidence of radiation cystitis in both Group-5.5 and Group-7.0 was 60%, which was higher than that in Group-6.0 (60% vs. 46.67%). In terms of radiation proctitis, the incidence of radiation proctitis in Group-5.5 was the highest (60%, 9/15), followed by Group-7.0 (33.33%, 5/15) and Group-6.0 (20%, 3/15). Notably, cases with radiation proctitis in Group-5.5 and Group-6.0 were all classified as Grade 2. However, in Group-7.0, one case developed Grade 4 radiation proctitis.

**Table 3:** Therapeutic efficacy and toxicitiy of different dose prescriptions in patients.

	Group-5.5	Group-6.0	Group-7.0	P value
**Local therapeutic efficacy**				>0.999
CR	13 (86.67%)	14 (93.33%)	14 (93.33%)	
PR	2 (13.33%)	1 (6.67%)	1 (6.67%)	
**Radiation cystitis**				0.804
Grade 0	6 (40%)	8 (53.33%)	6 (40%)	
Grade 2	9 (60%)	7 (46.67%)	9 (60%)	
**Radiation proctitis**				0.058
Grade 0	6 (40%)	12 (80%)	10 (66.67%)	
Grade 2	9 (60%)	3 (20%)	4 (26.67%)	
Grade 4	0	0	1 (6.67%)	

## Discussion

High-dose-rate brachytherapy is characterized by a shorter treatment duration and reduced occupational radiation exposure for medical staff. It has been shown to provide comparable or even superior outcomes compared to low-dose-rate brachytherapy (22, 23). Dose-fractionation schedules for brachytherapy vary significantly among different centers, and the optimal schedule is still a subject of debate (4–14). Therefore, our study aimed to investigate the dose-response relationships of different IGABT fractionation schedules in the treatment of uterine cervix carcinoma. In this study, although Group-5.5 achieved the highest HR-CTV and IR-CTV doses, it also exhibited the highest OAR doses in terms of bladder and rectum, thus resulting in poor clinical outcomes. Contrarily, Group-6.0 yielded a relatively high HR-CTV and IR-CTV doses with low OARs doses, providing the most beneficial clinical outcomes in brachytherapy for cervical cancer.

The study results revealed no significant positive correlation between the CR rate and the HR-CTV received dose during brachytherapy. For HR-CTV, the EQD_2_ corresponding to the brachytherapy dose of Group-5.5 was significantly higher than that of Group-6.0 and Group-7.0. However, in regard to the clinical therapeutic effects, the CR rate of Group-5.5 was lower than the CR rates of the other two groups. Even though the HR-CTV dose of Group-7.0 was the lowest, the CR rate of Group-7.0 was higher than that of Group-5.5. The lowest CR rate in Group-5.5 may be contributed by the longer overall treatment time compared to the other two groups. In the present study, the overall treatment time in Group-5.5, Group-6.0 and Group-7.0 was 59.47 ± 3.58, 56.40 ± 3.6 and 55.20 ± 6.74 days, respectively. Previous data show that patients were exposed with a 2.4-fold risk of local recurrence, and their local control rate decreased by 0.63% for each day, when the total treatment duration exceeded 8 weeks (24). Our findings aligned with these conclusions. It should be noted that the doses and numbers of fractions used in the three different dose-fractionation schedules (e.g., 5.5 Gy × 6 F, 6.0 Gy × 5 F, and 7.0 Gy × 4 F) in this study were all within the recommended range of ABS.

The dose of brachytherapy is determined by both the prescribed dose and the number of fractions. Generally, late-responding tissues tend to be more sensitive to the prescribed dose. A study by Patel et al. compared two dose-fractionation schedules, 9 Gy × 2 F (Arm A) and 6.8 Gy × 3 F (Arm B), revealing that the 3-year actuarial risks of developing Grade 3 and above toxicity in Arm A and Arm B were 7.47% and 3.57%, respectively (25). Similarly, Wang et al. demonstrated that the incidence of proctitis in the patients with 7.3 Gy × 3 F was higher than that of the patients with 4.8 Gy × 5 F (11); and this result is consistent with our finding that the incidence of radiation cystitis in Group-7.0 was higher than that in Group-6.0 (60.00% vs. 46.67%). At the same time, the incidence of radiation proctitis in Group-7.0 was higher than that in Group-6.0 (33.33% vs. 20.00%), and even one case developed Grade 4 radiation proctitis in Group-7.0. However, Le Pechoux et al. compared the dose-fractionation schedules of 5 Gy × 6F/4F (group A) and 6 Gy × 5 F/4 F/3 F (group B) showing that the incidence of side effects was 55% in Group A and 37% in Group B (26). Hsu et al. compared the dose-fractionation schedules of 7 Gy × 6 F and 8 Gy × 4 F, demonstrating that 8 Gy × 4 F caused fewer side effects than 7 Gy × 6 F (27). Dang et al. found that the incidence of Grades 2–3 rectal side effects was higher in patients who received the regimen with 6–7 fractions than in those who received 3–5 fractions (28). In our study, the incidence of radiation proctitis in Group-5.5 was the same as that in Group-7.0 and higher than that in Group-6.0 (60.00% vs. 46.67%). Moreover, the incidence of radiation proctitis in Group-5.5 was even higher than that in Group-6.0 (60.00% vs. 20.00%), and Group-7.0 (60.00% vs. 33.33%). During each brachytherapy, the positions of the bladder and rectum change to a certain extent, and the dose of brachytherapy drops rapidly, posing challenges in the accurate dose assessment of the OARs. Zhou et al. demonstrated that D_2cc_ was a predictor of rectal complications (29). Concerning the bladder, Kim et al. found that bladder D_2cc_ > 95 Gy could induce severe toxicity (30). In our study, the D_2cc_ value in the rectum was closely related to the incidence of radiation proctitis. However, no correlation was found between the D_2cc_ value in the bladder and the incidence of radiation cystitis.

If the prescribed dose is excessively high, the EQD_2_ based on the linear-quadratic formulation model may be overestimated due to dose saturation and the dynamics competition of chromosomal centromere (31). If the prescribed dose is too low, brachytherapy may be added early in the IMRT stage. Typically, tumor size reduction is not substantial in the early stage of IMRT, making the insertion of applicator a challenging aspect. Additionally, the proximity between the tumors and OARs is relatively short during this phase. On the premise of considering the received dose in OARs, the dose received by HR-CTV is far below the anticipated dose. The prescription dose and the number of fractions directly impact the HR-CTV and OARs dose. In clinical practice, it is crucial to carefully consider these two factors to find a balance, aiming to reduce the dose uncertainty in the target area and minimize the incidence of toxicity and side effects in OARs (e.g., the bladder and rectum).

In this study, no significant differences in the prognosis of the patients or the side effects were observed among our three dose-fractionation schedules. Except for HR-CTV D_90%_, HR-CTV D_98%_, and rectum D_0.1cc_, no significant differences in other dosimetric parameters were observed among the three schedules. The absence of statistical difference between the different groups may be due to the relatively small number of patients. Besides, the median follow-up time was only 30.08 months. Further research with a larger patient cohort and an extended follow-up period is crucial to validate these findings.

## Conclusions

The dose-fractionation schedule of 6.0 Gy × 5 fractions provided the most beneficial effects with relatively low OARs doses, suggesting that this dose-fractionation schedule should be prioritized in the clinical application of brachytherapy in cervical cancer.

## Data availability statement

The datasets presented in this article are not readily available because The raw data supporting the conclusions of this article will be made available by the corresponsding author with reasonable requests. Requests to access the datasets should be directed to 275232962@qq.com.

## Ethics statement

The studies involving humans were approved by the Ethic Community of Chongqing University Cancer Hospital. The studies were conducted in accordance with the local legislation and institutional requirements. The ethics committee/institutional review board waived the requirement of written informed consent for participation from the participants or the participants’ legal guardians/next of kin because of retrospective nature of the study.

## Author contributions

HW: Conceptualization, Formal analysis, Methodology, Writing – original draft, Writing – review & editing. YH: Data curation, Writing – review & editing. DC: Data curation, Writing – review & editing. ML: Data curation, Writing – review & editing. XZ: Conceptualization, Writing – original draft, Supervision, Writing – review & editing.
